# Prompt engineering in the age of generative AI: Insights from public discourse on social media

**DOI:** 10.1371/journal.pone.0351077

**Published:** 2026-06-11

**Authors:** Reuben Ng, Ting Yu Joanne Chow

**Affiliations:** Lee Kuan Yew School of Public Policy, National University of Singapore, Singapore; Kazan University, RUSSIAN FEDERATION

## Abstract

**Background:**

Public discourse on “prompt engineering” has grown rapidly, but large-scale evidence on how people discuss it is limited. The nascent coinage describes a user journey of optimizing text-based parameters within large language models and generative AI platforms in a trial-and-error process, adding modifiers or key phrases to yield a satisfactory output. While several studies have systematically and taxonomically mapped these techniques, no big data studies have specifically delved into the public reception to the concept. The study significantly contributes to the literature by documenting emerging new trends. It is prudent to keep abreast of discursive content as these techniques enter into the public lexicon, by capturing public discourse, sentiments and dominant themes.

**Objectives:**

This paper maps prominent discussion peaks and quarter-by-quarter themes and sentiment in posts mentioning “prompt engine*” on X and Reddit (1 May 2023–30 April 2024).

**Methods:**

We analyzed a large social media corpus (n = 298,774) of publicly-available English-language posts containing “prompt engine*” (engine/engineer/engineering/engineered). Our first research direction detected discussion spikes using peak-prominence modeling (pre-specified prominence threshold at 95^th^-percentile significance), then qualitatively coded the top-engagement posts on peak dates. Our second research direction computed quarterly sentiment, frequent bigrams, and emoji usage.

**Results:**

Six peaks centered on: (i) skill validity, (ii) future ramifications, (iii) title longevity, (iv) artist-attribution disputes, (v) model bias/guardrails, and (vi) analogies to child-rearing. Negative sentiment increased from 14.1% (Q1) to 37.3% (Q4), while positive sentiment fell from 36.4% to 22.8%. Tutorials dominated early content; later quarters featured humor, satire and legal liability discussions.

**Conclusions:**

Public narratives around prompt engineering increasingly question legitimacy, longevity, and ethics. Clearer terminology, transparency about guardrails, and guidance on responsible use may help align expectations and practice.

## Introduction

The rise and democratization of generative artificial intelligence, from text-based chatbots like *ChatGPT*, to text-to-image generators like *Stable Diffusion, Midjourney and DALL-E,* has permitted the public to use these models for personal and professional purposes. Individual users interact with these platforms by crafting prompts in a trial-and-error process [1], adding modifiers or key phrases to yield a satisfactory output [2]. This meticulous user journey has been coined as the process of “prompt engineering” [3]; spurring the publication of elicitation techniques and taxonomies [4–6], or circumventing model restrictions [7] in service of deriving the most ideal prompt. This methodology has promising applications in fields like medicine [8,9], programming [10], academia [11], informational literacy [12] and design uses [13]. While the emergent concept of “prompt engineering” has been interrogated for such professional applications, especially in the abovementioned best practices guides, publications have yet to capture the flurry of public discourses about this emerging concept.

This paper intends to fill this research gap in the context of a historic inflection point in artificial intelligence development. Capturing the dimension of social sentiments is crucial, given that that public support influences adoption of new technologies [14], and identifying dominant discourses and objections can help understand subjective topics [15] and streamline future implementation. Given the projected increasing integration of generative AI into the future of work [16,17], and the viability of “prompt engineer” as a professional skill [18] or profession [19], it is prudent to track conversational content surrounding prompt engineering’s entry into the public lexicon.

To achieve this, we analyze a corpus dataset of all English-language social media posts from X (formerly Twitter) and Reddit, containing “prompt engineering” as a keyword, in two research directions. First, we endeavored to evince the *biggest themes or discussions that generated the most social media engagement*, by means of running a prominent peaks model to identify key conversational drivers associated with mention count spikes (95^th^-percentile significance threshold of prominence>1,500). Second, we endeavored to chart the direction of conversations using the content metrics of sentiment valence scoring (positive, neutral, negative), highest-frequency bi-grams and emojis, on a quarter-on-quarter basis. By examining metrics that generated the most social buzz, we provide a macro perspective on narratives surrounding this topic, and an exploration into discussions on this emerging technology.

## Methods

### Dataset

**Fig 1:**
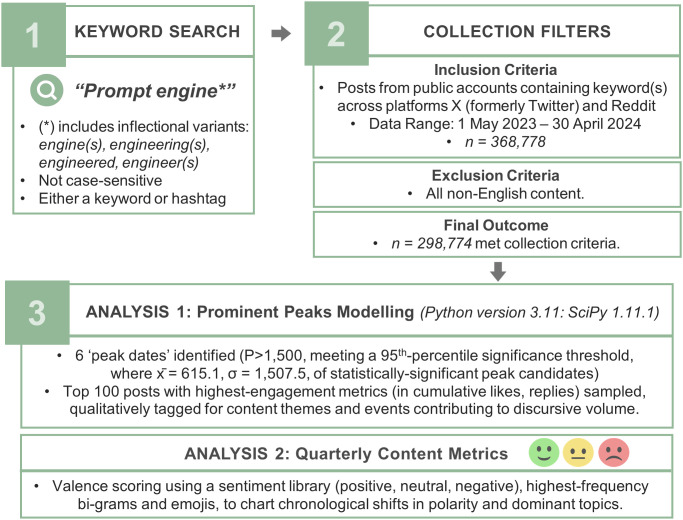
Methodological Summary: Data Collection and Analytic Plan.

The corpus was collected using X (formerly Twitter, using application programming interface standard search, publicly accessible via API V2 on the academic research access level) and Reddit (using Pushshift API, adhering to fair use non-commercial academic research). All English-language content containing “prompt engine*” as a keyword, including inflectional variants on “engine”, like engines, engineer(s), engineering, engineered, used in context, not case-sensitive, and either as a keyword or hashtag, posted within the span of a year (1 May 2023–30 April 2024) were collected (n = 298,774). A summary of the methodology is provided in Fig 1.

### Research Direction 1: Prominent Peak Modelling

Of our corpus dataset, daily mention counts (defined by the total number of original tweets, posts and replies containing the “prompt engine*” keyword) were charted. Using this data, prominent peak dates in which the keyword experienced a significant spike in its count were identified using peak prominence detection modelling package scipy.signal.find_peaks (Python version 3.11: SciPy 1.11.1). This methodology is attested in prior published papers charting burgeoning public perceptions of ChatGPT [20], using prominent peaks as a proxy signal for statistically-significant spikes in interest and engagement in the topic. The study design leverages emerging coinages to study novel phenomena and identify key drivers of buzz in a process of socially-derived agenda setting [21] and reflective of opinions circulating a societal microcosm [22].

The package module calculates for every date’s mention-count value (A), the lowest possible point prior to that date (B) and after that date (C): the average of these values (A-B; A-C) defined as the date’s prominence score, a value assigned relative to the lowest possible point on either side of the calendar. Of 97 candidates for statistically-significant peaks throughout the study period (x = 615.1, σ = 1507.5), prominence scores of said peaks ranged from 71 (lower quartile), 265 (median), 568 (upper quartile). A data-driven approach was used to determine an adequate threshold for significance: peaks that appeared within the 95^th^ percentile of prominence (i.e., prominence> 1,500) were selected for further investigation, yielding 6 significant peak dates. All posts published on these dates were then qualitatively interrogated by sampling the top-100 most-engaged posts (defined by an aggregated cumulative score of likes, replies, comments and reposts in the form of quotes) to identify the main content themes and discussions that led to an increment in online discussion. Thematic categorization was first conducted by two independent investigators, thereafter, reconvening to evaluate qualitatively-coded findings. Initial inter-coder agreeability was at 89.83%, while consensus-resolved agreement reached 96.67% of all sampled posts, with a remaining 3.33% of posts persisting in inter-coder nuanced disagreement, particularly for posts that tended to be about two topics at once. Investigators considered various conversational threads and opinions, taking care to note detractors (often in the form of rebuttal replies, or comments receiving high upvote volumes) and supporters (often in the form of agreeable replies, and large amounts of likes or upvotes) where applicable.

### Research Direction 2: Quarterly Conversational Metrics

The following content metrics used in conjunction with “prompt engineering” posts were amalgamated on a quarterly basis. First, each post was categorized as positive, neutral, or negative, with valence scoring using a fine-tuned transformer-based sentiment model roBERTa adapted from Google’s open-source pre-trained Bidirectional Encoder Representations from Transformer (BERT) for deep representations from unlabeled text through joint conditioning left and right context [23], most widely employed and cross-validated academically in the analysis of online sentiment analysis [24]. Alongside that, we present the highest-frequency bi-grams and top emojis per quarter. Top bi-grams were identified by raw count after text was tokenized into contiguous two-word sequences with the manual exclusion of stop-words, defined as auxiliaries containing little-to-no semantic information, for example, determiners, prepositions and conjunctions were omitted from qualifying for this list. Top emojis were identified by raw count attested in the dataset with reference to standardized Unicode emoji libraries and presented in order of highest count. This charts the shifting chronology in opinion, content foci, and prevailing social buzz surrounding this topic.

## Results

### Prominent Peaks and Emergent Themes

**Fig 2:**
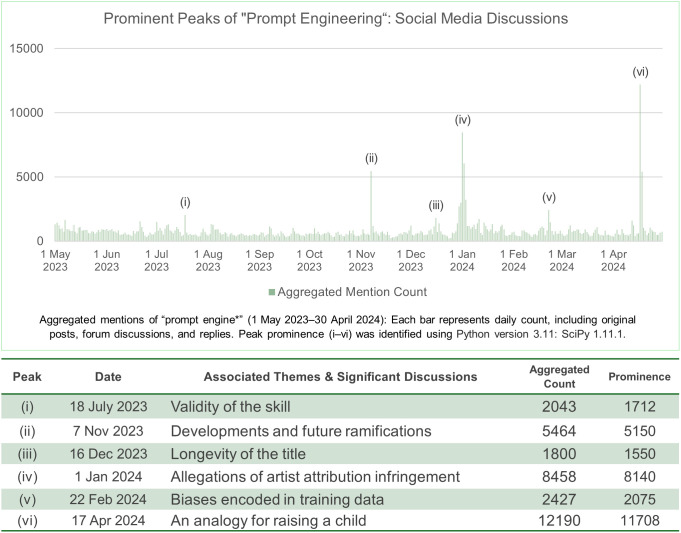
Prominent Peaks in “Prompt Engineering” Conversations across 300,000 Social Media Posts from 1 May 2023 to 30 April 2024.

A total of 6 prominent peak dates (i)–(vi) were identified and summarized in Fig 2, accompanied with a thematic discussion of each spike’s discourses.

### Peak (i): Validity of the Skill

This first peak date saw an uptick in discussions on the legitimacy of the title “prompt engineer”. Ongoing conversation saw differing opinions on the topic, consisting of three main opinion camps. First, detractors argued that prompt engineering was neither a real skill nor legitimate profession, and an over-complicated way of describing a simple process of typing text parameters into a large language model’s interface. Others mocked “prompt guides” produced by self-proclaimed “prompt gurus”, insinuating that the skill was “overhyped” to market productivity courses for personal profit. This camp pointed out that these platforms are themselves *already* a product designed for end users and did not require further hand-holding to use, as all it required was asking questions in English.

On the other hand, supporters believed that prompt engineering required legitimate training (e.g., precise word choices, streamlined questions, providing relevant code snippets, designing tasks as efficiently and clearly as possible, using fewer tokens to reduce confusion, identifying ideal prompt patterns, using “jailbreaks” or “prompt injections” with ingenuity to bypass filters) to elicit the best results (accurate responses, desired information, free from false information or “hallucinations”). Supporters highlighted problem formulation as an enduring and adaptable skillset, especially in knowledge-based jobs (e.g., finding information, assessing its viability, highlighting critical findings): fundamental components of the research workflow. Many pointed out that tech-illiteracy was still rampant in many industries, providing anecdotes of colleagues who were incapable of using search engines to find desired information, lacking the technological abilities to access basic tools, or unable to frame their search requests cogently: these disparities necessitating the skillset of the prompt engineer.

A third camp debated the semantics of “engineer”, perceiving that it was “pretentious” and an injustice to *actual* certified engineers, who had to complete a 4-year degree and receive proper accreditation to earn the title. This camp opined that more accurate titles should have been adopted, ranging from neutral ones like “prompter” or “prompt tweaker, crafter, specialist, developer or technician”; to denigrative, like “prompt monkey” or “code jockey”.

### Peak (ii) Developments and Future Ramifications

This discussion spike was attributable to several field updates. First, early access to “*PromptIDE*”, an “integrated development environment for prompt engineering and interpretability research” was announced by artificial intelligence company xAI. Second, artificial intelligence research organization OpenAI held their first developer conference “DevDay”, announcing custom GPTs with pre-customized prompts, made available for paid service users. These announcements generated discussions among AI enthusiasts, ranging from excitement over potential new tools, computational infrastructure and processing power; though other commenters tempered their expectations given that only a handful of screenshots and descriptions were included in the initial announcements. Other commenters suggested that these developments may affect small startups and independent bootstrappers dabbling in creating AI wrapper applications, given overlapping scopes. Others posited that with enough time and iterations, models would prove more capable than a human prompter in deciphering and crafting custom prompts, rendering the engineer’s role obsolete.

### Peak (iii): Longevity of the Title

This spike date occurred in conjunction with the circulation of OpenAI’s prompt engineering guide, containing strategies for obtaining optimal results. This spurred discussions on the usefulness of large language models, especially in coding and menial tasks, with the general consensus conceding that the technology had not lived up to its hype and initial promise of task automation and general all-purpose toolkit functionality. The viability of a prompt engineering “career” was also called into question: many pointed out the lack of job security and current limitations to the technology, suggesting that artificial demand created for the position would be phased out.

### Peak (iv): Allegations of Artist Attribution Infringement

This notable spike was attributed to a revelation that generative AI image-prompt program Midjourney developers were caught training their AI models on a database of 16,000 artists, whose artworks were scraped without consent. This information, submitted as evidence for an ongoing lawsuit, sparked outrage and condemnation at the blatant art theft. There was a clear discursive distinction made between individual human artists, who respectfully and organically learn from other inspiring artists, by replicating techniques to hone their skills to creatively express themselves, and self-proclaimed “AI prompt artists”, whose outputs were perceived as “lazy” and simply piggybacking off stolen amalgamated art. Many championed the use of novel digital tool “Glaze” [25] to sabotage future art scraping, suggesting that artists apply “style cloaks” before uploading their art online.

### Peak (v): Biases Encoded in Training Data

This peak date involved conversations on the transparency of in-house prompt engineers in shaping AI behavior, especially in fine-tuning models to reduce potential biases. Given that part of the data used to train models are drawn from the internet, an inherently “poisoned well”, containing unrestricted or unverified information, inevitably containing biases, hate speech, “polluted” with prejudices and stereotypes, discussions surrounded the need for high-quality training data, better curation and sanitization of large datasets to allow nuanced, respectful outputs authentically representing human society. However, this idealized workflow comes at the cost of being expensive, labor-intensive, and time-consuming.

Instead, commenters speculated that engineers were using pre-prompt instructions to correct for potential biases (e.g., adding keywords or removing sensitive words from the original prompt keyed into the user interface on the backend, blanket refusal of requests that may produce biased results). This method was perceived as “lazy”, “slapdash”, “ineffectual”, “wallpapers” over real problems, and only adds “token diversity” after the fact, given that the model still contains data pollution that can be easily circumvented using jailbreaking and prompt injection methods. Unintended consequences of being overly restrictive may surface (e.g., a parameter intending to encourage diversity may strongly emphasize minority representation and over-compensate by removing all other individuals from an output), suggesting a need for more guardrail transparency (i.e., documentation of guardrail topics, design ethos, specific filtering commands and code, reinforcement learning iterations and qualitative discussions on modifications made).

### Peak (vi): An analogy for Raising a Child

This biggest peak stemmed from an original tweet from OpenAI co-founder Elon Musk, who opined that raising children was akin to “18 years of prompt engineering”, which sparked thousands in engagement metrics. Some supporters of this analogy agreed that a brain was akin to a machine learning algorithm, given that artificial neural networks were conceptually modelled after neurons. On the other hand, more were of the opinion that child-rearing was *nothing* like prompt engineering, as it is a complicated social process involving years of teaching, guiding and inspiring a sapient being to learn and grow, intertwined with lessons on morality and appreciation for the world. Many took the opportunity to critique a larger societal narrative tied to Silicon Valley, pointing out the inhumane oversimplification of comparing raising a child to a ChatGPT session and the irrationality of “tech bros” who speak about childcare like running diagnostics on a robot.

### Content Metrics

**Fig 3:**
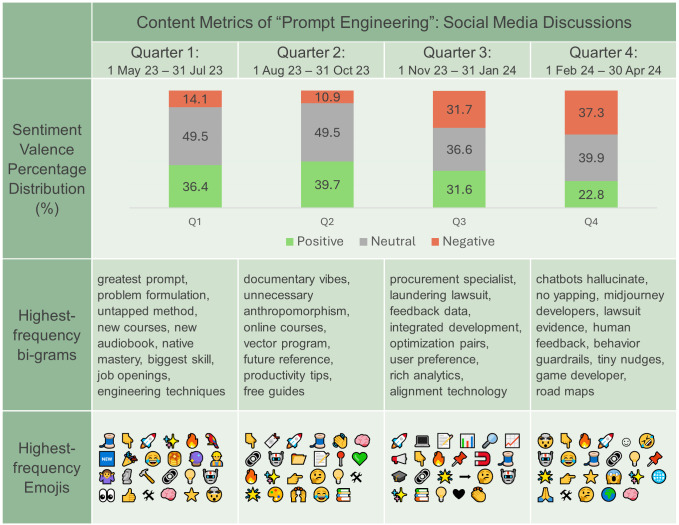
Content Metrics of ‘Prompt Engineering’ Conversations across 300,000 Social Media Posts from 1 May 2023 to 30 April 2024.

Salient content metrics were identified on a quarter-on-quarter basis (wherein each quarter’s keyword count is as follows: Q1 n = 73,326, Q2 n = 53,444, Q3 n = 93,074, Q4 n = 78,930) and summarized in Fig 3 and elaborated further in 5 key findings. Finding 1 highlights shifts in valence scoring, finding 2 highlights predominant themes encoded in highest-frequency bi-grams and emojis embedded in tutorial posts, and findings 3–5 elaborate on high-frequency bi-grams containing notable conversational buzz topics surrounding prompt engineering discussions.

### Finding 1: Increasingly Negative Valence

Over the study period, the negative valence of “prompt engineering” posts increased from 14.1% in Q1 to 37.3% in Q4. Conversely, positivity percentages decreased from 36.4% in Q1 to 22.8% in Q4. Overall, this suggests waning positive sentiments toward the field over the course of a year.

### Finding 2: Predominant Tutorial Posts

Throughout the dataset, there was a marked prevalence of posts marketing prompt engineering tutorials, courses, guides, and individuals championing the field as “the next big thing”. Of the highest-frequency bi-grams, a majority contained an educational slant. For instance, bi-grams connoted to informative material (“new courses”, “new audiobook”, “online courses”, “free guides”), best techniques (“biggest skill”, “untapped method”, “problem formulation”, “engineering techniques”) and the potential impact on career and work (“job openings”, “productivity tips”). Overall, such posts advertised resources purported to transform one’s efficiency, reflecting a broad surge in the circulation of prompt-engineering guides online.

Similarly, highest-frequency emojis conveyed informative sources (**🧵, 🔗, 📂, 📝, 📚, 🎓, 🧠, 🔖, 📢:** the symbols of the thread, hyperlink, file folder, memo, books, academic cap, brain, bookmark and megaphone respectively), technological potential (**🚀, 🤖, 🌐, 🌍, 💻, 📊, 📈**: symbols of the rocket, robot, world wide web, globe, laptop and statistical charts respectively), call-to-action pointers toward cardinal directions (**👇, 👉, ➡**), toolkit (**🔨, 🛠, 👷, 🔎**: symbols of the hammer, construction tools, construction worker and magnifying glass respectively), various expressions of excitement and positivity (**🆕, ✨, ⭐, 🌟, 🔥, 🎉, 🔮, 💡, 👍, 👏, 🙌, 🤯**: symbols of newness, stars and sparkles, party popper, crystal ball, lightbulb, thumbs up, applause, raised hands celebrating success, mind-blown respectively). Several instances of doubt were displayed in emojis like **🤔** (thinking face), **🤷** (shrug), **😂🤣** (laughing-crying symbols), **😱** (fear).

### Finding 3: Multiple Instances of Humor

Present in Q1 was the bi-gram “greatest prompt”. The context of the original post had a mocking tone, with the poster claiming to be the “greatest prompt engineer” as they had prompted ChatGPT to rank U.S. presidents on their “absorbency” and managed to circumvent the chatbot’s initial resistance to answering the question given its absurdity and political nature. Through the use of hypothetical scenario prompting, the output ranked the absorbent quality of George Washington’s “powdered wig” against Theodore Roosevelt’s “robust moustache”. The post garnered 9.3 thousand likes and was an example of users deliberately eliciting humorous replies using prompt engineering while engaging with large language models.

Also present in Q2 was the bi-gram “documentary vibes”. The context of the original post mocked generative AI users for claiming to be “artists” when all they did was type words into an interface using such prompts as “award winning photograph”, “beautiful Rembrandt lighting”, “extreme fine art photography (SUBJECT) in the style of (PHOTOGRAPHER)”. The crux of the commentary hinged on whether such self-proclaimed “art prompt engineers” were displaying artistry, or simply “stealing” from the work of established artists. The post garnered 20 thousand likes and was an example of users discussing the ethics behind artistic integrity.

Present in Q4 was the bi-gram “no yapping” (vernacular for being excessively verbose). The context of the original post jokingly suggests that this phrase was the perfect “pro-level” prompt engineering strategy, contrary to an existing litany of overly-complex rules that prompt engineer “gurus” tout online. The poster mocks existing guidelines and subverts the expectation of precise prompt construction with the use of colloquial slang. The post received 15 thousand likes and was an example of users poking fun at the current prompt engineering landscape, pointing out the latent absurdity of taking the field too seriously.

### Finding 4: Legal Implications of Using AI Chatbots

Present in Q3 was the bi-gram “procurement specialist”. The context of the original post was an individual conversing with a car dealership’s AI Assistant, demonstrating how simple it was to use specific prompts to get the chatbot to make promises the company could not honor. Specifically, the original post claimed to feed prompts into the chat window to obtain a car for $1.00 USD, managing to receive an affirmative response that read “*that’s a deal, and that’s a legally binding offer*”. The chat bot was later deactivated. The post garnered 101 thousand likes, emblematizing the ease at which bots could be manipulated using specific prompts to promise consumers items they had no apparent authority over.

Similarly, present in Q4 was the bi-gram “chatbots hallucinate”. The context of the original post cited a recent international airline having to honor a policy invented by its chatbot after it provided inaccurate information on bereavement refunds to a passenger. The poster jokingly suggests for other passengers to use prompt engineering to make airline chatbots hallucinate in a similar way to offer “$1 first class tickets”. The inciting post sparked discussions on the ease at which consumers could elicit perks using prompt loopholes, and whether corporations should be responsible for ensuring information across their AI chatbots or webpage assistants was accurate to avoid potential financial liabilities.

### Finding 5: Stylistic Impacts

Present in Q2 was the bi-gram “unnecessary anthropomorphism”. The context of the original post claimed that the process of prompt engineering was akin to a keyword search in a library, and that prompters ought not to erroneously imagine they are engaging with a human when using large language models. This post invited discussions on the validity of this claim, given that such models were trained on natural language written by humans and for humans, thus making it valuable to converse with it like a human. For instance, adding “please” and “thanks” to prompts, or using compassionate directives like “take a deep breath and proceed to work on this step-by-step” were alleged to produce better results, lending credence to simulating anthropomorphism when framing tasks linguistically. The long-term implications on linguistic mannerisms in online writing were also surfaced, with some reporting that large language model ChatGPT had an identifiable uncanny “accent”: often too polite (e.g., using too many phatic tokens, being tonally-obsequious or sycophantic, prone to empty flattery), framing answers vaguely and avoiding voicing critique toward the prompter.

## Discussion

This paper analyzed large-scale data on public discussions, focusing on prominent engagement themes and content metrics surrounding the emergent concept of prompt engineering. Our findings from our first research direction revealed 6 key peaks, revealing that social buzz was most focused whether the skill was foundationally-valid, or merely a short-lived fad. Conversations circulated around the semantic significance of “engineer”, longevity of the AI bubble, and social issues like artist copyright, societal biases encoded in training data, and what it means to be human and raise a child, compared to training a large language model. Our findings from our second research direction revealed that sentiment valence has grown increasingly negative over the course of a year, with posts about guidebooks and productivity tutorials giving way to multiple popular posts that use humor to mock the “prompt guru” ecosystem, the legal implications of using unreliable AI chatbots, and the long-term impacts of language models on the linguistic styles in online writing. Taken together, whether or not the concept of prompt engineering stands the test of time or evolves into viable career options, this foray surfaced discussions that are deeply philosophical, ethically complex, and occasionally humorous; broad lessons with wide applicability as humanity continues grappling with the concept of artificial intelligence and its place in our lives.

The abovementioned trends corroborate comparable social media studies on emerging AI technologies, where other features like generative AI have similarly resulted in negative sentiment trends in the context of creative and art-based professions [26], suggesting that such concerns and public perceptions are far-reaching and require careful integration into society. Similarly, other studies on engagement patterns regarding public reception to LLMs [20] reflect double-edged excitement over powerful new tools and wariness toward misuse. The current literature points to persistent skepticism and engagement commonly driven by debates over authenticity and authorship, with users actively developing “AI-free” labels to signal human effort. These findings are particularly relevant in the context of the technology acceptance model [27], wherein current developments in generative artificial intelligence have rapidly become integrated into the private and professional lives of users, yet whether or not such technologies are adopted or rejected depends on social perceptions and emotional influences. The observed shifts in sentiment and peak-related patterns therefore point to initial points of hesitancy that challenge whether such technologies will be widely accepted or face societal pushbacks in the long run. Further, these findings may be contextualized against existing work in communication studies and the social sciences, wherein theories on agenda-setting [28] could meaningfully explain the disproportionate and heightened public engagement in specific topics at certain peak points in time. On social media, this amplification effect is observable in bursts of heightened engagement, where algorithmic trending and rapid sharing create a feedback loop that places a specific topic at the forefront of collective consciousness. Thus, this exploratory work provides an overview of collective attention placed on this topic of technological adoption, facilitated by the architecture of digital platforms.

This study is limited in its generalizability in several ways. First, findings are limited to English-language posts, thereby omitting discourse from different cultural and social dimensions. Future research may consider incorporating data from non-English languages (e.g., Spanish, Chinese, Arabic, etc.) to reveal additional nuance. Second, data was collected across two social media platforms, X and Reddit. Future studies may incorporate data from other platforms (e.g., TikTok, YouTube, LinkedIn, Instagram, etc.) to provide a more comprehensive picture of public responses to the emergent concept of prompt engineering.

Also inherent to the nature of data collection methodology are potential biases such as demographic skew, wherein the findings of the study may only be applicable to typically younger users of social media sites, or populations with easier access to such platforms. Furthermore, the study’s selection of the top 100 highly-engaged posts for each peak highlights the most popular perspectives, but may exclude low-like but meaningful contributions, which could introduce a bias in the results. The use of sentiment analysis on social media text as a research metric may also unintentionally misclassify emotional tone, given that no model can authoritatively assert user intent with full confidence. These findings are also limited in being descriptive, focusing on exploring observed patterns and temporal trends, rather than making inferential claims about their underlying societal drivers.

Separately, the dataset may be subject to potential social manipulation: conversational content may contain influence by bot activity or organized campaigns, particularly by individuals or organizations with a vested interest in promoting the concept as a marketable skill [29]. Additionally, as the dataset was constructed using predefined search strings, posts that discuss the topic indirectly or use alternative terminology could be omitted, potentially limiting completeness and introducing systematic exclusion of certain perspectives or contexts.

Future research may track whether this concept has gained traction over time or fades into relative obscurity, further charting public perceptions as changes are made in the AI landscape. Specifically, the methodology used in this study may be replicated for the same keywords over subsequent time periods, alongside emergent keywords relevant to this topic [30]. Conceptually, this paper builds on a structured, replicable methodology that interrogates social narratives of explosive new phenomena. Practically, this study deepens our understanding of how society reacts to new and evolving technological advancements [31], charting dominant discourses that may be used to inform tech companies messaging and framing strategies with future updates.

## Supporting information

S1 Data Dataset. (XLSX)
